# Radiomics predicts the prognosis of patients with clear cell renal cell carcinoma by reflecting the tumor heterogeneity and microenvironment

**DOI:** 10.1186/s40644-024-00768-7

**Published:** 2024-09-16

**Authors:** Ji Wu, Jian Li, Bo Huang, Sunbin Dong, Luyang Wu, Xiping Shen, Zhigang Zheng

**Affiliations:** 1 Department of General surgery, Suzhou Ninth Hospital Affiliated to Soochow University, Suzhou, Jiangsu Province China; 2 Department of Radiology, Suzhou Ninth Hospital Affiliated to Soochow University, Suzhou, Jiangsu Province China; 3 Department of Radiology, Changshu No People’s HospitalThe Affiliated Changshu Hospital of Nantong University, Changshu, Jiangsu China; 4 Department of Radiology, Municipal Hospital Affiliated to Nanjing Medical University, Suzhou, Jiangsu Province China; 5 Department of Radiology, Renji Hospital, School of Medicine, Shanghai Jiao Tong University, Shanghai, China

**Keywords:** Overall survival, Clear cell renal cell carcinoma, Deep learning, Radiomics, Tumor microenvironment

## Abstract

**Purpose:**

We aimed to develop and externally validate a CT-based deep learning radiomics model for predicting overall survival (OS) in clear cell renal cell carcinoma (ccRCC) patients, and investigate the association of radiomics with tumor heterogeneity and microenvironment.

**Methods:**

The clinicopathological data and contrast-enhanced CT images of 512 ccRCC patients from three institutions were collected. A total of 3566 deep learning radiomics features were extracted from 3D regions of interest. We generated the deep learning radiomics score (DLRS), and validated this score using an external cohort from TCIA. Patients were divided into high and low-score groups by the DLRS. Sequencing data from the corresponding TCGA cohort were used to reveal the differences of tumor heterogeneity and microenvironment between different radiomics score groups. What’s more, univariate and multivariate Cox regression were used to identify independent risk factors of poor OS after operation. A combined model was developed by incorporating the DLRS and clinicopathological features. The SHapley Additive exPlanation method was used for interpretation of predictive results.

**Results:**

At multivariate Cox regression analysis, the DLRS was identified as an independent risk factor of poor OS. The genomic landscape of different radiomics score groups was investigated. The heterogeneity of tumor cell and tumor microenvironment significantly varied between both groups. In the test cohort, the combined model had a great predictive performance, with AUCs (95%CI) for 1, 3 and 5-year OS of 0.879(0.868–0.931), 0.854(0.819–0.899) and 0.831(0.813–0.868), respectively. There was a significant difference in survival time between different groups stratified by the combined model. This model showed great discrimination and calibration, outperforming the existing prognostic models (all p values < 0.05).

**Conclusion:**

The combined model allowed for the prognostic prediction of ccRCC patients by incorporating the DLRS and significant clinicopathologic features. The radiomics features could reflect the tumor heterogeneity and microenvironment.

**Supplementary Information:**

The online version contains supplementary material available at 10.1186/s40644-024-00768-7.

## Introduction

Renal cell carcinoma (RCC) is one of the most deadly urological malignancies [1, 2]. The most common subtype of RCC is clear cell renal cell carcinoma (ccRCC), which accounts for the majority of RCC-related deaths [3]. Radical nephrectomy and nephron-sparing surgery are the primary treatments for localized ccRCC, but the prognosis of ccRCC patients varies among different stages [4]. It is challenging to accurately predict the clinical outcome because of tumor heterogeneity.

To the best of our knowledge, several prognostic models have been proposed for outcome prediction of localized ccRCC after surgery [5–7]. For example, the Stage, Size, Grade, and Necrosis (SSIGN) score and the University of California, Los Angeles, Integrated Staging System (UISS) have been validated and widely used in clinical setting [8, 9]. However, most of these studies were limited to the clinicopathologic level and their predictive performances were unsatisfactory. What’s more, the characteristics of intratumor heterogeneity were not considered in these studies. A more comprehensive prediction could be an intriguing topic for further investigation.

Contrast-enhanced computed tomography (CT) examination is mandatory for detailed assessment of the nature of renal mass [10]. In recent years, a newly established field, converts extracted features into quantitative parameters [11–13]. This method shows huge potential in disease diagnosis and prognosis prediction [14]. However, the association of CT-derived DL radiomics features with clinical outcomes in ccRCC patients remains unclear. We hypothesized that DL radiomics features could provide valuable information for outcome prediction.

Last but not least, previous studies revealed that radiomics features could reflect the tumor heterogeneity [15, 16]. However, the link between imaging subtypes and tumor microenvironment remained unexplored. We aimed to investigate and visualize this relationship.

Overall, we aimed to explore the prognostic implications of CT-derived DL radiomics features, and then generate a prediction model for OS in ccRCC patients. The secondary endpoint was to explore the associations between DL radiomics features and tumor heterogeneity/ microenvironment.

## Methods and materials

### Study design

This retrospective multicenter cohort study has been reported in line with the STROCSS criteria [17]. We strictly followed the ethical guidelines of the 1975 Declaration of Helsinki. The Research Ethics Committee of Suzhou Ninth Hospital Affiliated to Soochow University had approved it.

**Fig. 1:**
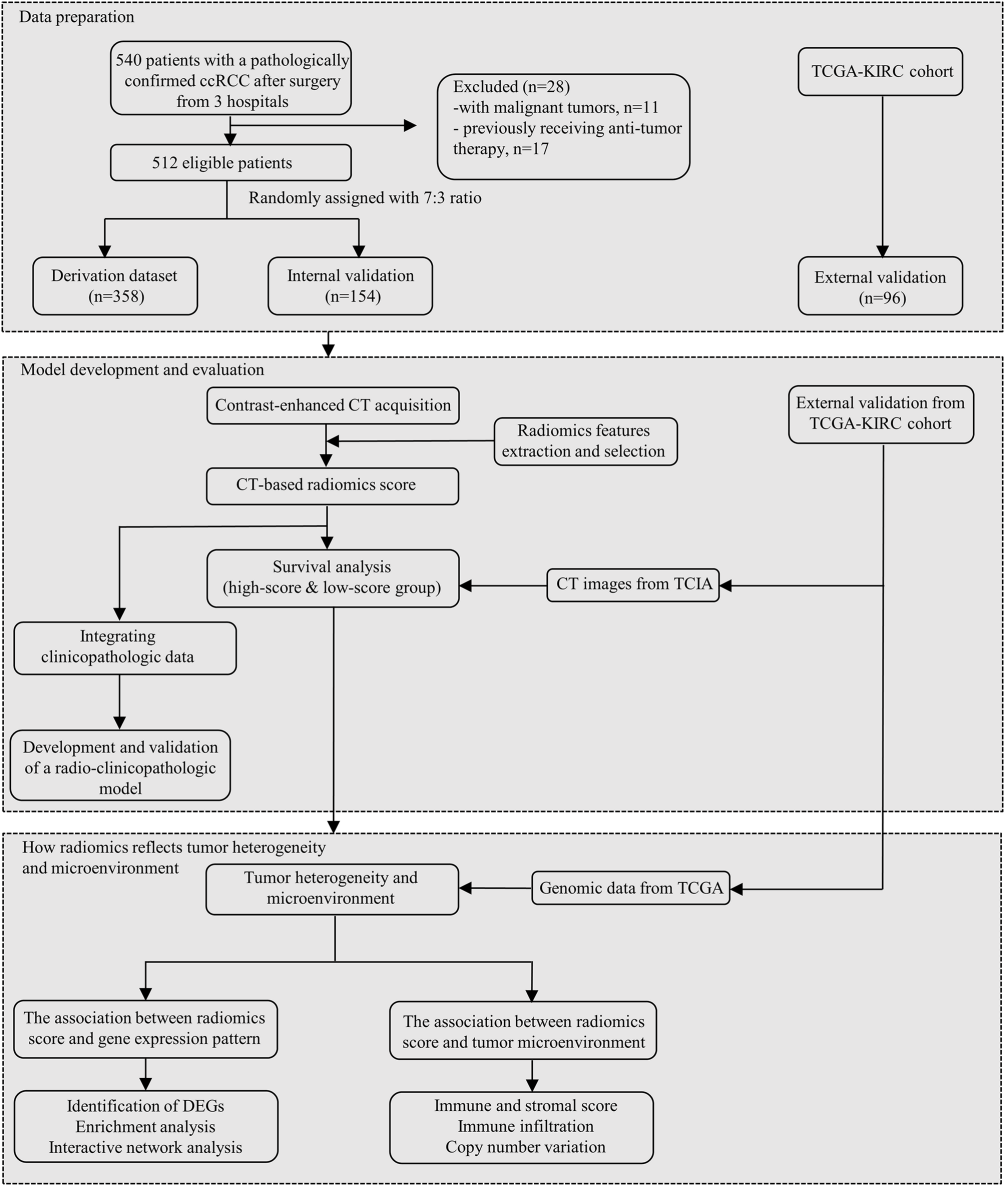
Flow chart of the study design. *Abbreviation ccRCC* clear cell renal cell carcinoma; *TCGA* the Cancer Genome Atlas; *TCIA* the Cancer Imaging Archive; *KIRC* kidney renal clear cell carcinoma; *DEG* differential expression gene

Localized ccRCC patients from 3 hospitals between January 2003 and July 2023 were recruited (Fig. 1). Clinical data were collected from the electronic medical record. The inclusion criteria were (1) older than 18-year-old (2) pathologically confirmed ccRCC after radical or partial nephrectomy (3) complete clinicopathological data, follow-up data and contrast-enhanced CT images. Patients with other malignant tumors or history of receiving anti-tumor therapy were excluded. We randomly divided the whole cohort into two groups (i.e., derivation and test cohorts) for model development and validation, with a ratio of 7:3 by the method of random number table.

### Primary outcomes

All individuals were followed up every 3 months for the first year and every 6 months thereafter by telephone. The primary outcome for this study was overall survival (OS), which is defined as the period between the date of diagnosis and death or the last follow-up. The secondary outcome was disease-free survival (DFS), defined as the time from primary surgery to first tumor recurrence, progression, death or the last follow-up.

### Definitions of potential predictive factors

Eastern Cooperative Oncology Group Performance Status (ECOG-PS) is a questionnaire for functional status assessment. This score showed close correlation with cancer mortality [18].

Charlson Comorbidity Index (CCI), a widely used scoring system, quantifies comorbidities based on the number and severity of diseases [19].

The prognostic role of the microvascular invasion has been reported in ccRCC [20, 21].

World Health Organization/International Society of Urologic Pathologists (WHO/ISUP) classification was used for pathological grading of RCC [22].

### Image acquisition and ROI segmentation

Contrast-enhanced CT images were collected from picture archiving and communication system. CT scan protocols are listed in Supplementary Table S1. Contrast-enhanced CT images and detailed clinical information of the external validation cohort (TCGA-KIRC cohort) were collected from The Cancer Imaging Archive (TCIA) (https://www.cancerimagingarchive.net/) [23]. The exclusion criteria were described as follows: (1) no enhanced CT images (2) poor image quality (3) survival time < 30 days. As a result, 96 cases were enrolled. Genomic data matching the TCIA were downloaded from TCGA (https://portal.gdc.cancer.gov/).

3D slicer software (Version 4.11.0) was used for the segmentation of region of interests (ROIs) [24]. The tumor area was outlined slice-by-slice by two experienced radiologists (10-and 15-years’ experience in radiology, respectively) using the “Level Tracing” function of the 3D Slicer. Areas comprising air and non-tumor tissues were manually removed. The boundary is smoothed using the “Smooth” function. The dispute on the region of interest (ROI) delineation would be settled after discussion. The largest cross-sectional slice of the 3D-ROI was selected as the input image for the ResNet50 model. This ROI area was extended outward into a square area. For the image standardization, we resampled all images to a 1 × 1 × 1 mm voxel spacing and performed gray-level discretization. In the gray-level discretization processing, CT images were set to soft tissue window (Window Width:350, Window Level:50), followed by mapping to the grey scale range [0, 255] (bin width, 25; bin count, 11).

Two experts reviewed and manually labelled CT images when blind to clinical information. The intra- and inter-class correlation coefficients (ICCs) were adopted for evaluating the intraobserver and interobserver reproducibility of the DL radiomics features [25]. In our study, 50 patients’ CT images from the derivation cohort were selected at random for ICCs calculation twice, in order to ensure the reproducibility of radiomics analysis.

### Feature extraction and selection

The transfer learning framework was introduced to overcome the challenge of small sample size. We extracted the DL radiomics features from the ROIs using the pre-trained classification model ResNet50 (Python 3.6, TensorFlow 2.0.0, Keras 2.3.1). The parameter combinations were listed as follows: activation=’ReLu’, optimizer=’Adam’, classification function=’sigmoid’, learning rate=’0.01’ and epoch = 100. We recorded training and validation loss values, and the corresponding network weights for epochs. During convergence, early stopping was used to prevent overfitting. The final model was determined for radiomics feature extraction, with the highest accuracy and lowest loss value.

The features with ICCs ≤ 0.8 were dropped. Then, we adopted the Spearman correlation coefficient (Rho) to evaluate the correlation between any two CT image features. If two features are highly correlated (i.e., |Rho| > 0.8), either of the two features would be excluded. Next, the univariate Cox analysis was conducted to identify OS-related features. Only features with p value < 0.05 were retained for further analysis. Finally, the least absolute shrinkage and selection operator (LASSO) Cox regression was performed to remove unimportant features. The deep learning radiomics score (DLRS) was then calculated based on weights of their respective coefficients.

### Predictive performance and prognostic value of the DLRS

A time-dependent receiver operator characteristic curve (ROC) was used to assess the predictive performance of the DLRS. The optimal cutoff value was calculated based on the maximum of Youden index. Patients were stratified into high- and low-score groups according to the best cutoff. Kaplan-Meier survival curves for OS and DFS were plotted respectively. In addition, the generalization of the DLRS was validated using the CT images from the TCIA (TCGA-KIRC cohort).

### Gene set enrichment analysis between high- and low-score groups

We collected CT scans and genomic data from the TCIA (TCGA-KIRC cohort) for revealing the molecular mechanism associated with the DLRS differences. Patients were grouped into high- and low-score groups by the DLRS. Differentially expressed genes (DEGs) were identified between both groups based on the R software “limma” package, with the thresholds of adj p value < 0.05 and |log_2_FC| > 2 [26].

DEGs were subjected to cell component, molecular function and biological processs studies by Gene Ontology (GO) analysis and Kyoto encyclopedia of Genes and Genomes (KEGG) pathway analysis [27, 28]. Both p and adj p values are set to less than 0.05.

STRING (the Retrieval of Interacting Genes, https://string-db.org/) is web tool to evaluate the potential relationship among these screened DEGs, with a confidence score of ≥ 0.9 [29]. Additionally, survival data from TCGA-KIRC cohort were used to evaluate the prognostic value of DEGs.

Finally, tumor mutation burden (TMB) refers to the sum of gene mutations in tumor cells [30]. In general, tumors with higher TMB may be more likely to respond to immunotherapy. Correlations between DEGs expression and TMB were investigated in our study.

### The association of the DLRS with tumor microenvironment

Firstly, the Estimate method was used for estimating tumor purity, stromal score and immune score in high- and low-score groups [31, 32]. Secondly, the CIBERSORT tool was applied to infer the infiltrating immune cells in ccRCC [33, 34]. The immunophenotype differences between different radiomics score groups were analyzed. Thirdly, we compared the immune cell infiltration levels among tumors with different somatic copy number alterations by using the Mann–Whitney U test [35, 36]. TIMER tool, a web server for comprehensive analysis of tumor-infiltrating immune cells, was used to visualize the distributions of each immune subset at each copy number status in ccRCC [35].

### Development and validation of the combined model

Clinicopathological data were collected from the electronic medical record including age, gender, body mass index, ECOG-PS, CCI score, TNM stage, tumor size, tumor necrosis, histologic grade, microvascular invasion, laboratory tests and clinical symptoms (hematuria and flank pain).

Univariate and multivariate Cox regression analysis were used to identify independent risk factors of poor OS. In the derivation cohort, eXtreme Gradient Boosting classification (XGBC) algorithm was introduced to generate a combined model by incorporating the DLRS and significant clinicopathological features [37]. The best parameters combination and eligible features were obtained by the method of 10-fold grid-search. We plotted the learning curve for fine-tuning (e.g., n_estimators). The final model with the highest accuracy was determined for the downstream analyses. (Python, version 3.6; scikit-learn package, version 0.24).

Time-dependent ROC analysis was conducted to evaluate the predictive performance. The clinical utility and stability of this combined model were evaluated by decision curve (DCA) and calibration curve analysis [38]. For visualizing the contributions of each feature to outcome prediction, Shapley Additive exPlanations (SHAP) plot was used to explain the impact of selected features on predictions [39].

Furthermore, SSIGN and UISS scores were commonly used for the assessment of long-term prognosis in ccRCC patients in clinical practice. The methods for SSIGN and UISS calculation can be obtained in Supplementary S2.

### Statistical analysis

All data analysis were performed with softwares (SPSS, version. 26.0 or R software, version. 4.0). Continuous data was shown as the median ± interquartile range (IQR). The comparison of continuous variables was conducted by the Mann–Whitney U test or Student’s t test. Categorical variables were compared by the chi-square test.

The ICCs were adopted for evaluating the intraobserver and interobserver agreement.

The Spearman correlation coefficient (Rho) was used to evaluate the correlation between variables. We compared the immune cell infiltration levels by using the Mann–Whitney U test. Time-dependent ROC analysis was performed to evaluate the predictive performance of models. Delong test was performed to compare the statistical significance between ROC curves. Univariate and multivariate Cox regression analysis were adopted to screen for independent risk factors of poor OS. The Kaplan–Meier method and log-rank test were used to estimate the survival.

We estimated sample size using pmsampsize package of R software (R2cs = 0.27, parameters = 10, prevalence = 0.15), and then at least 296 cases were required for model development [40]. All significant tests were 2- sided and p values < 0.05.

## Results

### Patient characteristics

**Fig. 2:**
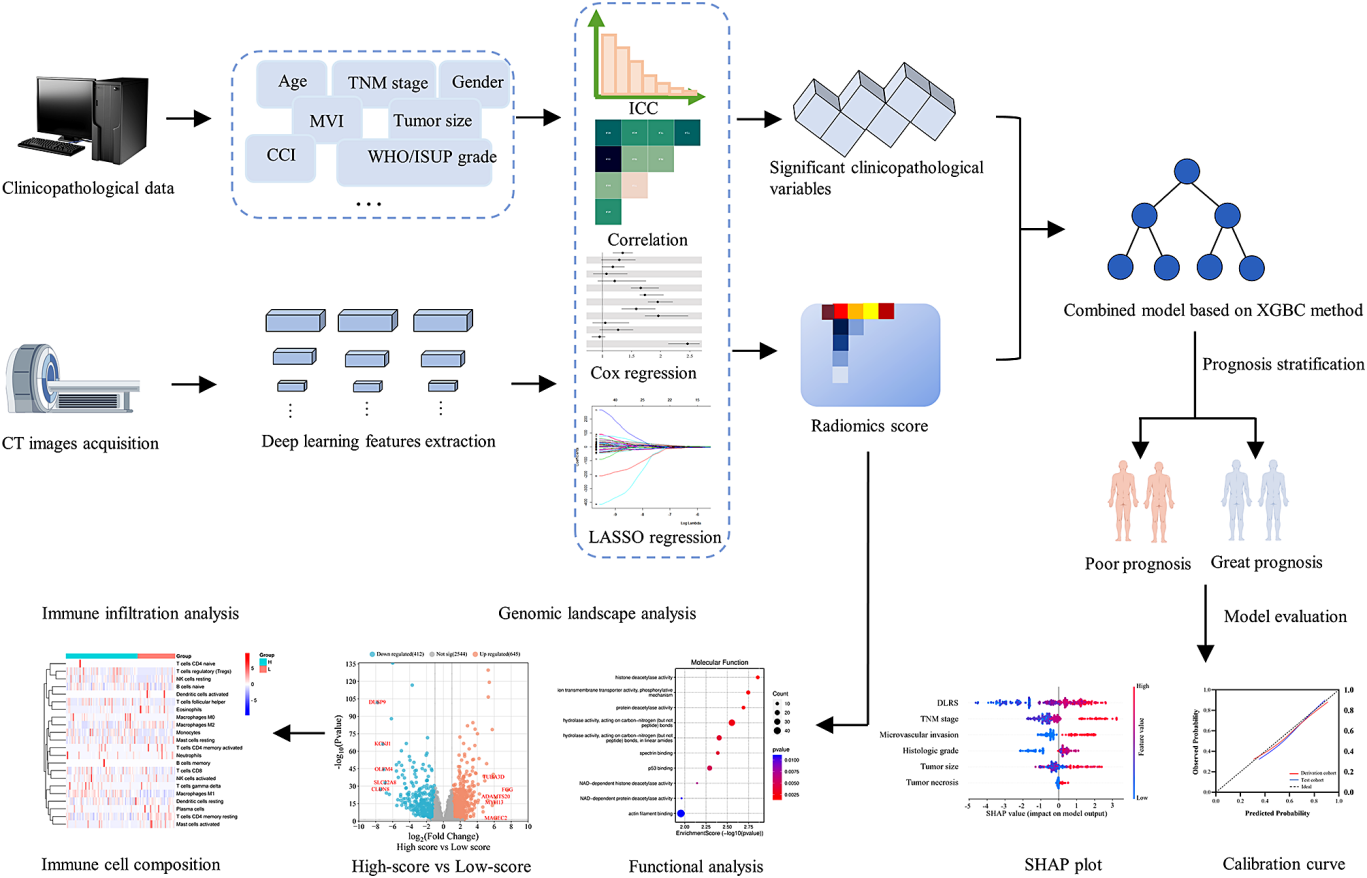
Technical flow chart of the study. *Abbreviation CCI* Charlson Comorbidity Index; *MVI* microvascular invasion; *WHO/ISUP* World Health Organization/International Society of Urologic Pathologists; *ICC* intra- and inter-class correlation coefficients; *LASSO* least absolute shrinkage and selection operator; *XGBC* eXtreme Gradient Boosting classification; *SHAP* SHapley Additive explanation

The overall study design is demonstrated in Fig. 2. A total of 512 eligible patients from 3 hospitals were recruited. 358 of 512 patients were randomly assigned to the derivation cohort. The remaining 154 cases and 96 cases from TCGA-KIRC cohort were reserved for model validation. Finally, a novel combined model, namely NHSTM-R, has been developed by integrating the DLRS [R] and independent clinicopathological features (N [tumor necrosis], H [histologic grade], S [tumor size], T [TNM stage] and M [microvascular invasion]).

The details of patient characteristics are shown in Table 1. In the whole cohort, the 5-year OS and DFS rates were 67.9% and 61.2%, respectively. Median OS was 62.2(IQR, 25.3) months for the derivation cohort, 59.7(IQR, 26.8) months for the test cohort. Median DFS was 54.3(IQR, 24.1) months for the derivation cohort, and 52.5(IQR, 23.7) months for the test cohort. In addition, only the OS time of patients in the TCGA cohort could be available, and the median OS was 70.4(IQR, 28.5) months.

**Table 1:** Patient characteristics

Characteristics	Derivation cohort *n* = 358	Test cohort	*p* value
		*n* = 154	
Age, year	59 (15)	58 (12)	0.751
Male gender	236(64.8)	92(59.7)	0.433
BMI, kg/m^2^	22.3(3.1)	23.1(2.6)	0.517
ECOG-PS (0/1/2) ^a^	191/164/3	88/66/1	0.396
CCI score ^b^	7(6)	7(5)	0.754
Hematuria	65(18.2)	15(9.7)	0.016
Flank pain	57(15.9)	25(16.2)	0.682
Tumor size, cm	5.1(4.3)	5.6(3.5)	0.267
Tumor necrosis	24(6.7)	13(8.4)	0.363
TNM stage			0.472
Stage I	284(79.3)	117(76)	
Stage II	29(8.1)	16(10.4)	
Stage III	42(11.7)	19(12.3)	
Stage IV	3(0.9)	2(1.3)	
Histologic grade			0.395
G1	42(11.7)	16(10.4)	
G2	211(58.9)	87(56.5)	
G3	95(26.6)	47(30.5)	
G4	10(2.8)	4(2.6)	
Microvascular invasion			
Laboratory tests			
Platelet count, 10^9^/L	234(75)	271(89)	0.051
Hemoglobin, g/L	127(31)	116(28)	0.152
Serum calcium, mmol/L	2.63(0.27)	2.46(0.21)	0.202
Creatinine, umol/L	86(22)	74(19)	0.185
Mean OS time, month	62.2(25.3)	59.7(26.8)	0.364
Mean DFS time, month	54.3(24.1)	52.5(23.7)	0.252

Quantitative values are median (IQR) and categorical variables are n (%)

^a^ ECOG-PS is a questionnaire to assess the functional status

^b^ CCI score is a widely used comorbidity scoring system and quantifies comorbidities based on the number and severity of diseases

*Abbreviation BMI* body mass index; *ECOG-PS* Eastern Cooperative Oncology Group Performance Status; *CCI* Charlson Comorbidity Index

### The DLRS calculation and evaluation

Of the 3566 DL radiomics features, 2781 reproducible features were identified based on ICC analysis. Next, of the 2781 features, 1753 DL radiomics features were retained by using the Spearman rank correlation test. Then, 297 DL radiomics features with p value < 0.05 were retained based on the univariate Cox analysis. Finally, by using the LASSO Cox regression method, we selected 7 most valuable DL radiomics features for the DLRS calculation.

DLRS = 0.517*DL_751-0.235* DL_1064 + 0.624* DL_1389 − 0.195* DL_1686 + 0.741* DL_1891 − 0.438* DL_2016 + 0.366* DL_2458 − 0.930.

In the test cohort, the DLRS had a great performance, with AUCs for 1, 3, and 5 year-OS of 0.814(95% CI, 0.775–0.859), 0.796(95% CI, 0.761–0.846) and 0.782(95% CI, 0.749–0.827), respectively (Table 2).

In the TCGA-KIRC cohort, the DLRS also had a great performance, with AUCs for 1, 3, and 5 year-OS of 0.775(95% CI, 0.742–0.801), 0.759(95% CI, 0.735–0.793) and 0.751 (95% CI, 0.730–0.778), respectively.

**Table 2:** Predictive performance of the models for overall survival prediction in patients with clear cell renal cell carcinoma

Performance	AUCs(95%CI)		
	For 1-year OS	For 3-year OS	For 5-year OS
Derivation cohort			
DLRS	0.839(0.782–0.871)	0.806(0.752–0.863)	0.787(0.749–0.846)
NHSTM-R	0.903(0.876–0.964)	0.862(0.822–0.905)	0.845(0.817–0.883)
SSIGN score	0.764(0.701–0.817)	0.752(0.695, 0.796)	0.729(0.674–0.751)
UISS score	0.722(0.648–0.764)	0.697(0.621–0.749)	0.648(0.592–0.706)
Test cohort			
DLRS	0.814(0.775–0.859)	0.796(0.761–0.846)	0.782(0.749–0.827)
NHSTM-R	0.879(0.868–0.931)	0.854(0.819–0.899)	0.831(0.813–0.868)
SSIGN score	0.745(0.702–0.809)	0.766(0.723–0.803)	0.717(0.654–0.749)
UISS score	0.725(0.656–0.758)	0.708(0.633–0.758)	0.663(0.614–0.729)

*Abbreviations OS* overall survival; *DLRS* deep learning radiomics score; *NHSTM-R* a combined model integrating the DLRS and independent clinicopathological features(tumor necrosis, histologic grade, tumor size, TNM stage and microvascular invasion); *SSIGN* Stage, Size, Grade, and Necrosis; *UISS* University of California, Los Angeles, Integrated Staging System; *UISS* University of California, Los Angeles, Integrated Staging System ; *AUC* area under of ROC curve; *CI* confidence interval

**Fig. 3:**
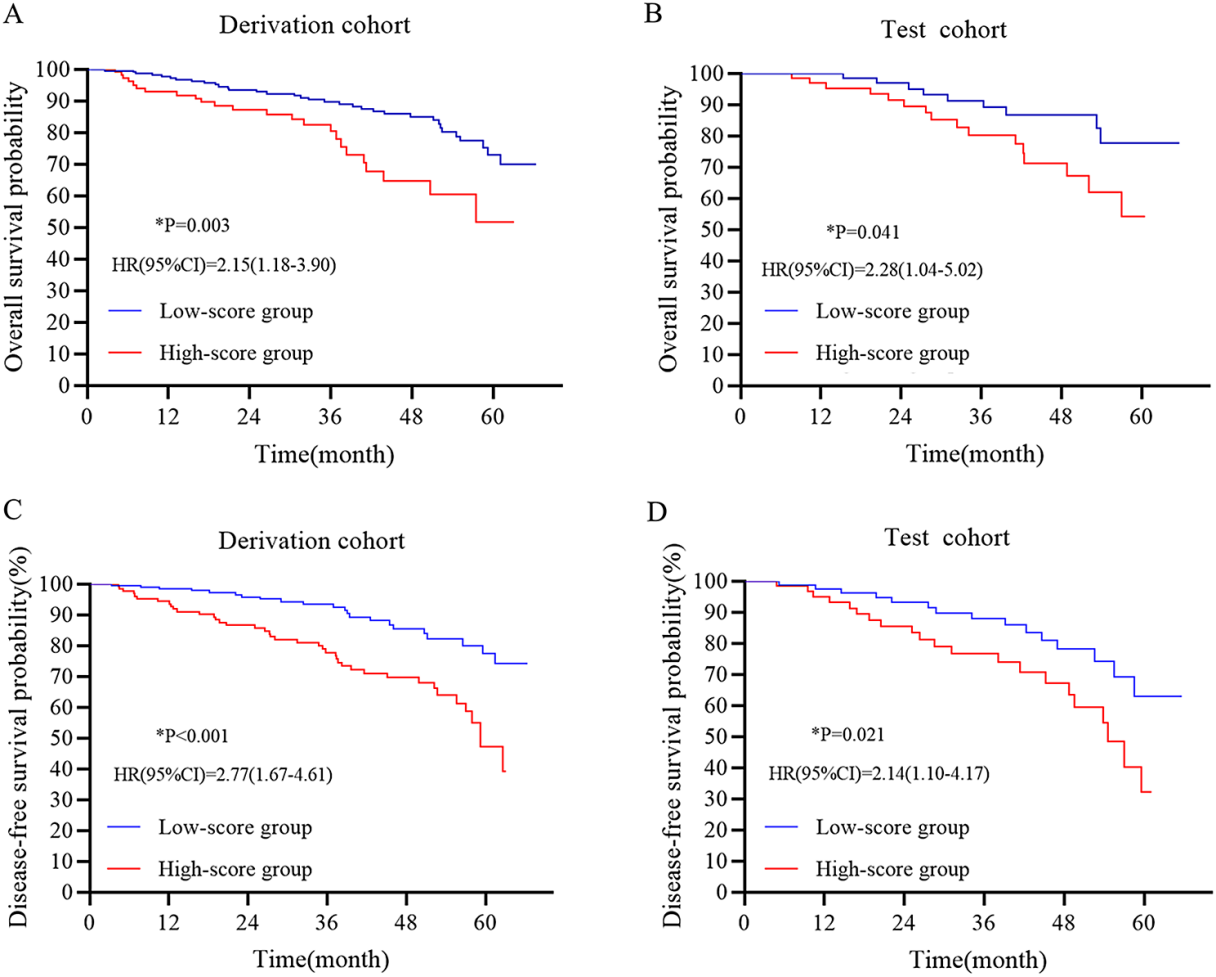
Kaplan-Meier survival curves for overall survival (**A**, **B**) and disease-free survival (**C**, **D**) in the derivation and test cohorts. Patients were stratified into high- and low -score groups by the deep learning radiomics score. Red line represents patients with high score and blue line represents patients with low score. We calculated p values using the log-rank test and results revealed that patients in high-score group were prone to poor prognosis (p values < 0.05 for all)

Patients were classified into high- and low-risk groups on the basis of the best cutoff value (-36.0162). Of the derivation and test datasets, 121 and 47 individuals were classified into the high-risk group, and 237 and 107 individuals were classified into the low-risk group, respectively. Kaplan–Meier survival analysis revealed that ccRCC patients in low-score group had a better OS and DFS compared to those in high-score group (all p values < 0.05) (Fig. 3A-D). The prognostic value of the DLRS was also determined in the external validation cohort (Supplementary Figure S1).

### Genomic differences between high- and low-score groups

**Fig. 4:**
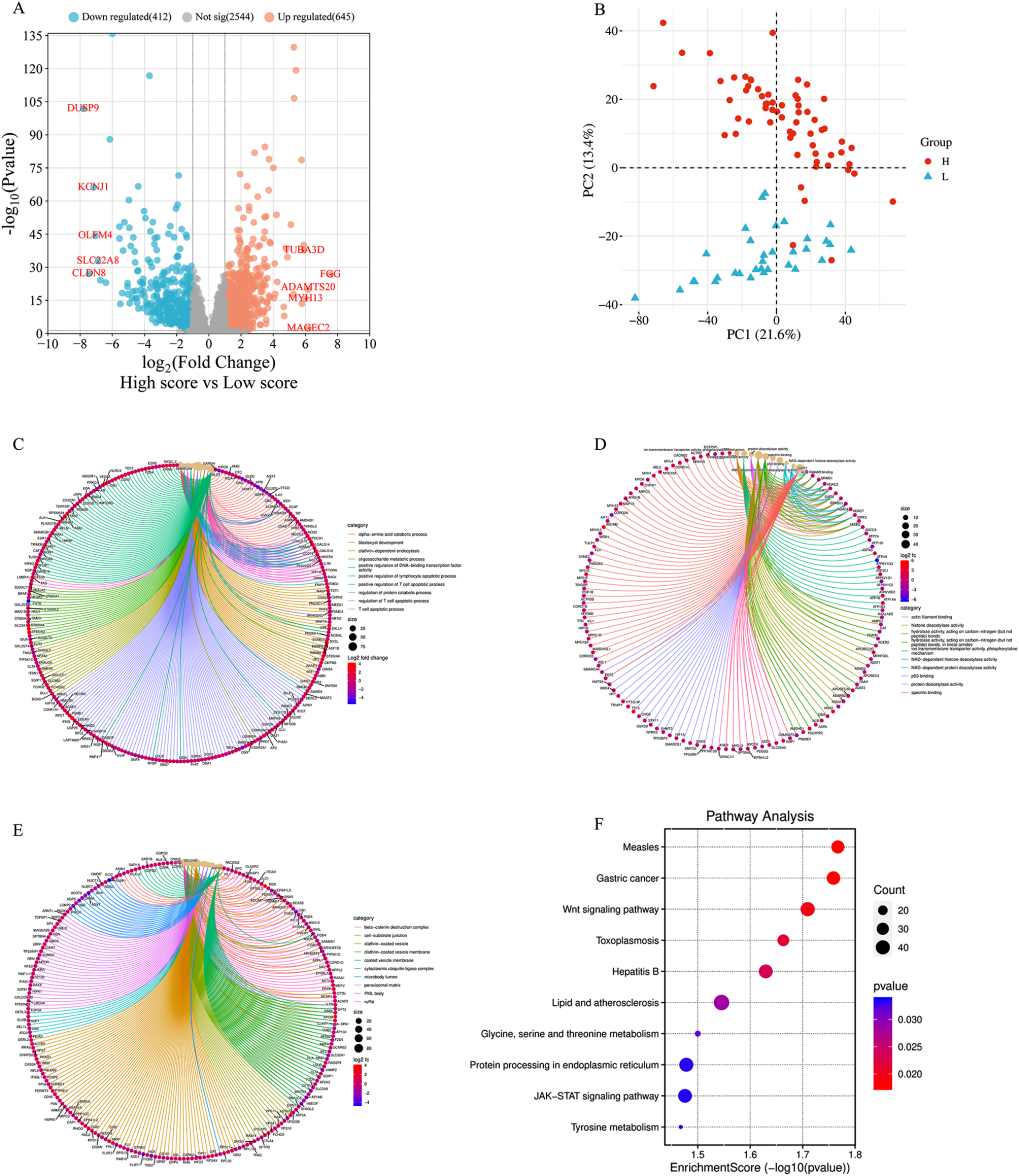
The association between radiomics score and gene expression patterns. (**A**) DEGs are shown in the volcano plot. (**B**) factor map of the PCA performed on 96 tissue samples and all DEGs. Two cluster groups were identified corresponding to low-score group (blue) and high-score group (red). GO annotation and KEGG pathway enrichment analyses were performed in DEGs. (**C**) biological process, (**D**) molecular function, (**E**) cellular component, and (**F**) KEGG analysis. The color scale indicates different thresholds of the p value, and the size of the dot indicates the number of genes corresponding to each pathway. *Abbreviation DEG* differential expression gene; *PCA* principal component analysis

To further explore the genomic differences between high- and low-score groups, we determined DEGs using RNA-seq data from TCGA cohort, and then generated a volcano plot showing 260 upregulated DEGs and 187 downregulated DEGs (Fig. 4A). Principal components analysis (PCA) revealed significant differences in gene expression level between both groups (Fig. 4B). GO analysis showed that DEGs were enriched in the biological process of immune system regulation: mainly regulation of T cells and lymphocyte (Fig. 4C-E). KEGG analysis indicated that DEGs were intensively enriched in Wnt signaling pathway and JAK-STAT signaling pathway, which were consistent with the findings of previous studies [41–44] (Fig. 4F). Interaction network analysis among DEGs was visualized in Supplementary Figure S2.

To investigate the association of DEGs with prognosis in ccRCC patients, prognostic value of the 10 most significant DEGs were analyzed. We observed that most of DEGs were significantly related to the OS (Supplementary Figure S3). In addition, the correlations between DEGs expression and TMB were visualized with radar chart (Supplementary Figure S4). CLDN8 and KCNJ1 gene expression levels showed significantly negative correlation with TMB (both p values < 0.05).

### The association of the DLRS with tumor microenvironment

**Fig. 5:**
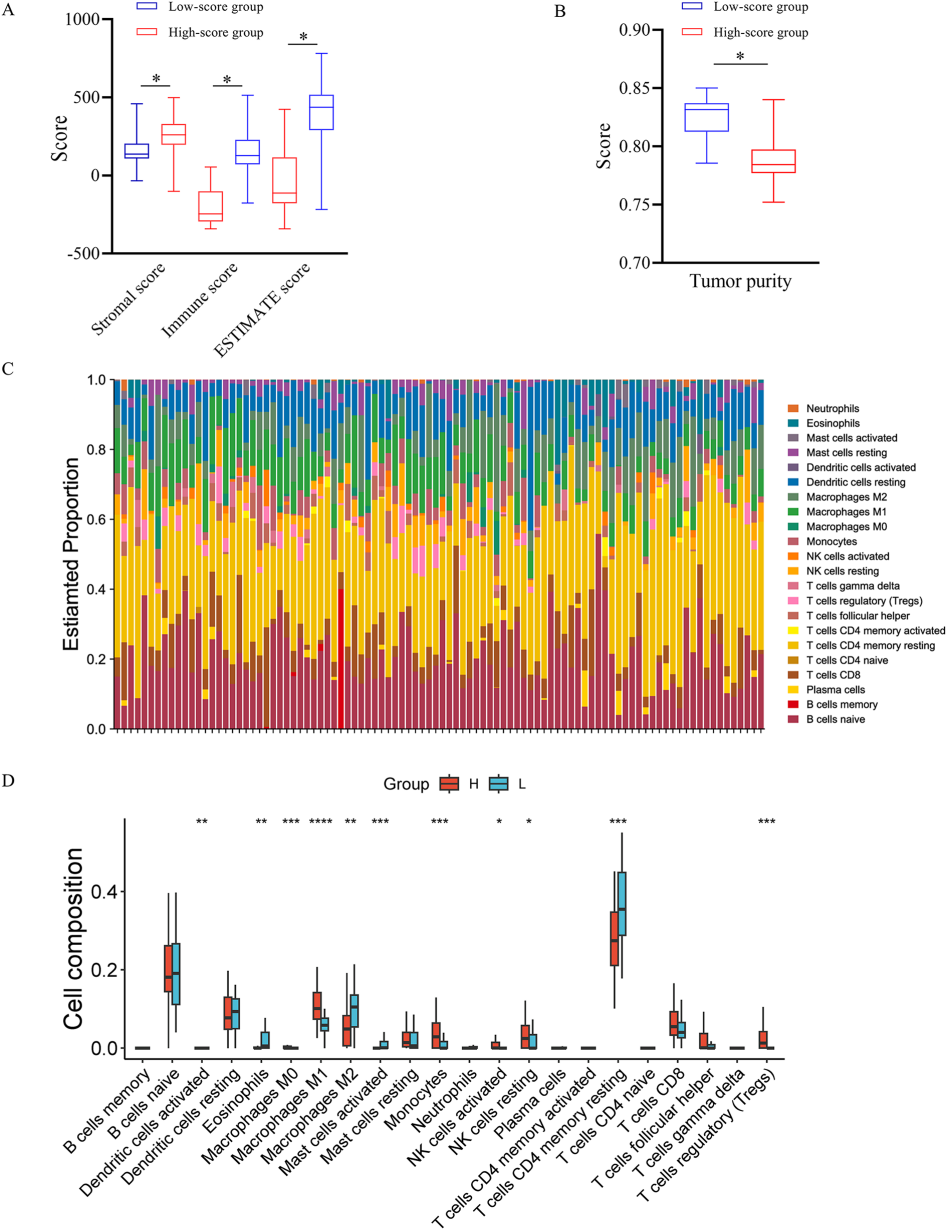
The association between radiomics score and tumor microenvironment. There were significant differences in stromal score, immune score, ESTIMATE score (**A**) and tumor purity (**B**) between high- and low-score groups. The fractions of 22 subsets of immune cells were analyzed and different colors indicate different immune cells (**C**). 63 samples on the left belong to high-score group, and 33 samples on the right belong to low-score group. The boxplot shows the difference in immune infiltration between high- and low-score groups (**D**). The horizontal axis indicates 22 immune cells and the vertical axis indicates cell content. The individuals with high score are labeled in red, and those with low score are labeled in blue

Firstly, the immune score, stromal score, ESTIMATE score (the sum of immune and stromal scores) and tumor purity were calculated, and significant differences were observed between high- and low-score groups (Fig. 5A, B). This indicated that radiomics score could reflect the immune infiltration levels. Secondly, since multiple immune-related biological processes were enriched, CIBERSORTx was used to calculate the relative proportion of 22 immune cell types of each case (Fig. 5C). We observed that multiple immune cells such as macrophage and regulatory T (Treg) cells varied significantly between different radiomics score groups (Fig. 5D). Finally, box plots were presented to show the distributions of each immune subset at each somatic copy number status of DEGs (Supplementary Figure S5).

### Identification of independent risk factors

**Fig. 6:**
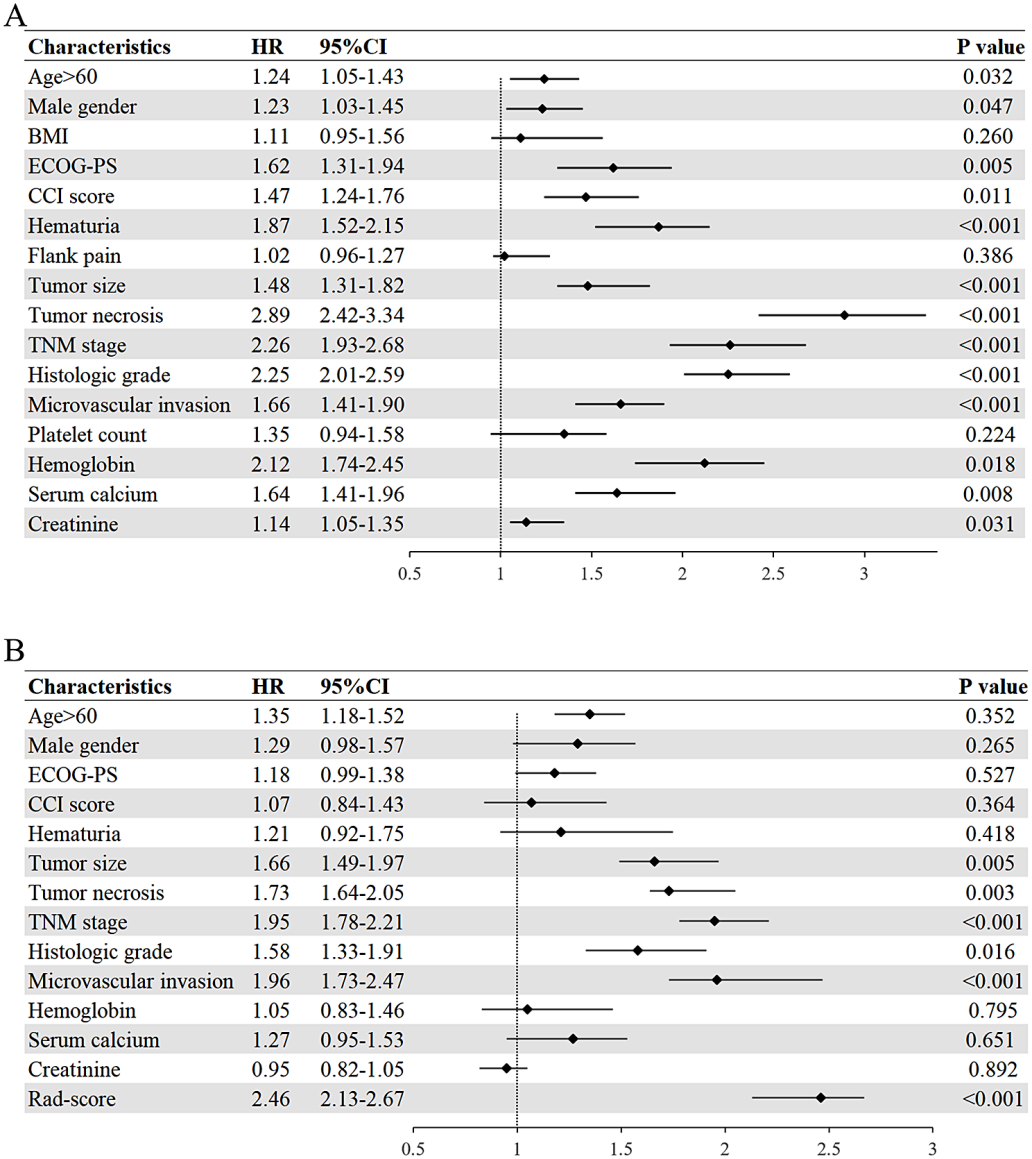
Forest plots for the univariate (**A**) and multivariate (**B**) Cox regression analysis, describing the association between each feature and poor overall survival. The vertical line represents the value of no effect. Data are presented as the HR value with 95%CI. p values are tested by Cox proportional hazard model. *Abbreviation HR* hazard ratio; *CI* confidence interval

Univariate Cox regression analysis results are displayed in Fig. 6A. In multivariate Cox regression analysis, the tumor size (HR, 1.66), tumor necrosis (HR, 1.73), TNM stage (HR, 1.95), histologic grade (HR, 1.58), microvascular invasion (HR, 1.96) and DLRS (HR, 2.46) were identified as independent risk factors of poor OS (Fig. 6B).

### Combined model construction and validation

**Fig. 7:**
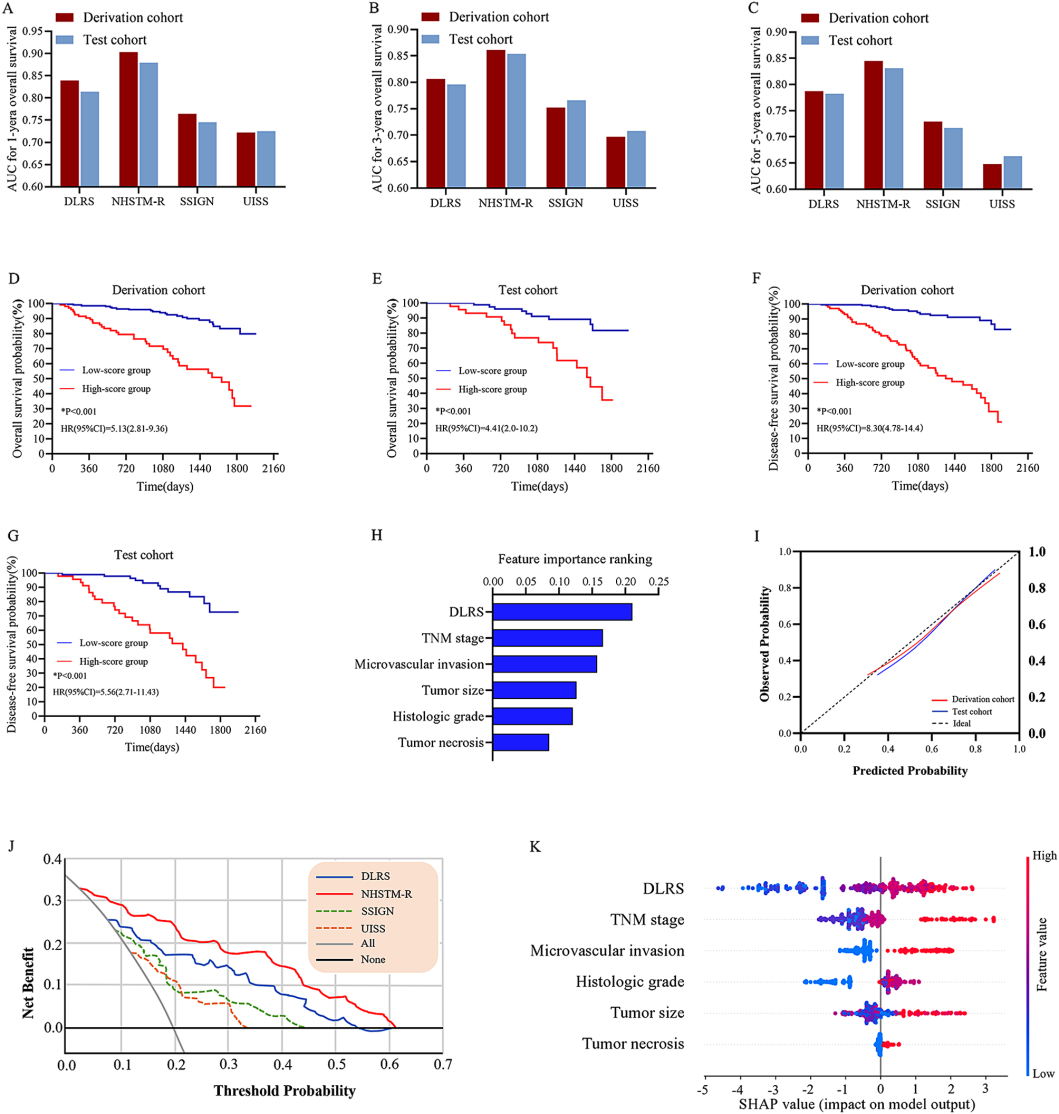
The predictive performance of the models. (**A**-**C**) the AUCs of four models for predicting 1, 3 and 5 year-OS were calculated in the derivation and test cohorts. (**D**-**G**) patients were stratified into high- and low -score groups by the combined model (namely NHSTM-R). Kaplan–Meier survival curves of the high- (red line) and low-score (blue line) groups showed significant differences. (**H**) features importance ranking. (**I**) calibration curves of the NHSTM-R model in the derivation and test cohorts. (**J**) decision curve analysis revealed that the NHSTM-R model showed great clinical utility. (**K**) SHAP summary plot explained the detailed contribution of features to prediction at the global level. The colors represent the magnitude of the features values and vary from high to low. Each point on the plot represents a particular feature of an individual. Y-coordinate is determined by the features that the point represents. X-coordinate is determined by the feature’s impact on the model’s output. *Abbreviations AUC* area under the curve; *DLRS* deep learning radiomics score; *SSIGN* the Stage, Size, Grade, and Necrosis score; *UISS* the University of California, *Los Angeles* Integrated Staging System; *HR* hazard ratio; *SHAP* SHapley Additive explanation

A combined model (namely NHSTM-R) was built based on the DLRS and independent clinicopathological features. The optimal parameter combinations of the NHSTM-R model are described in Supplementary S3. In the test cohort, the NHSTM-R model had the AUCs for 1, 3, and 5 year-OS of 0.879(95% CI, 0.868–0.931), 0.854(95% CI, 0.819–0.899) and 0.831(95% CI, 0.813–0.868), respectively (Table 2). Delong test revealed that the NHSTM-R model significantly outperformed the DLRS and existing prognostic models (all p values < 0.05) (Fig. 7A-C). Kaplan-Meier survival curves for OS and DFS are shown in Fig. 7D-G. There was a significant difference in survival time between different radiomics score groups stratified by the NHSTM-R model.

Furthermore, the feature importance rankings were calculated (Fig. 7H). The NHSTM-R model was well calibrated in both cohorts, and had a larger net benefit than the others in the whole cohort (Fig. 7I, J). As shown in SHAP summary plots, we visualized the contributions of each feature to OS prediction by using the average SHAP values (Fig. 7K). We found that the DLRS acted as a pivot role in the outcome prediction.

## Discussion

In the present study, we generated and externally validated the DLRS for predicting the OS in ccRCC patients. By using multi-omics data from TCGA-KIRC cohort, we revealed the tumor heterogeneity and microenvironment between different radiomics score groups. Furthermore, we developed a novel combined model (namely NHSTM-R) for OS prediction by incorporating the DLRS and independent clinicopathological features. This model showed great discrimination, calibration and clinical utility. SHAP analysis was adopted to help clinicians better understand the predictive results.

Intratumor heterogeneity (ITH), a key driver of tumor progression, can be exhibited on the radiological level [45]. DL techniques can autonomously acquire feature representations of ITH from medical image data on the basis of artificial neural networks [46]. Consequently, these techniques open up a broad scope of future research in the field of disease diagnosis and prognosis prediction for RCC patients. The estimation of OS is important for individualized management of ccRCC patients [5]. Only a few studies have applied DL methodologies to prognosis prediction in ccRCC patients [25, 47, 48]. Nie et al. reported that the DL radiomics model was developed for predicting cancer-specific survival in localized ccRCC patients [47]. This model showed favorable predictive performance. However, the potential value of clinicopathological data has not been explored in their study. In our study, multivariate Cox analysis showed close association between the radiomics score and OS. Schulz et al. also reported that DL radiomics features could stratify ccRCC patients [49].

To the best of our knowledge, previous studies have explored gene expression patterns in different image subtypes. Wang et al. revealed the association between the gene expression level and radiomics features in breast cancer [15]. A few studies have provided insights into that how radiomics reflect the heterogeneity of ccRCC and tumor microenvironment. He et al. and Wang et al. establish CT-based radiomics models for predicting prognosis-related genes expression for ccRCC patients by using CT images [16, 50]. Consistent with above two studies, our study found that lipid metabolism was significantly associated with image subtypes. Meanwhile, Wnt and JAK-STAT signaling pathways were also enriched, and GO analysis for cell component indicated that DEGs were particularly enriched in β-catenin destruction complex which is the heart of the canonical Wnt signaling pathway [51]. Reportedly, WNT signaling pathway played a vital role in the proliferation and self-renewal of cancer stem cells in ccRCC [41]. It has been reported that multiple genes such as CD56 polysialylation and CENPA had great impacts on tumor progression and metastasis via the Wnt/β-catenin signaling pathways in ccRCC [42, 52, 53]. Similarly, JAK-STAT pathway was found associated with the ccRCC progression and OS [43, 54, 55].

Notably, GO analysis for biological process revealed that T cell and lymphocyte apoptotic processes were also enriched in the present study, which suggested that there were significant differences in the tumor microenvironment between image subtypes. This implied that the DL radiomics features could reflect the heterogeneity of tumor microenvironment. The tumor microenvironment plays a key role in the progression of ccRCC. Liu et al. determined the prognostic value of infiltrating immune cells within the tumor microenvironment in ccRCC, and illustrated the underlying mechanism by which infiltrating immune cells promoted cancer progression [56]. What’s more, the close correlation between the radiomics features of ccRCC and tumor-infiltrating immune cells was observed as it is claimed in a previous study [16].

In this study, we also found that tumor purity and the proportion of immune cells significantly varied between high- and low-score groups (p value < 0.05). Macrophage and Treg cells were higher in the high-score group (p value < 0.05), and could be promising targets for cancer immunotherapy. Farha et al. reported that a cluster of ccRCC patients defined by enrichment in M0 macrophages had poor prognosis [57]. Xu et al. reported that ccRCC patients with high Macrophage-M1 fractions had worse prognosis than those with low Macrophage-M1 fractions [58]. Yang et al. reported that SGOL1 promoted ccRCC cell proliferation and invasion by increasing the Treg cells infiltration [59]. In additional, we observed that patients in high-score group had more CD8 T-cell infiltrates compared to those in low-score group (p value > 0.05). Giraldo et al. reported that CD8 + T cells showed positive correlation with unfavorable prognosis in ccRCC [60]. Overall, the poor prognosis of ccRCC patients in high-score group could be caused by the extent of immune cell infiltration. This tumor microenvironment could be reflected by the radiomics features.

Furthermore, clinicopathological features such as TNM stage and histologic grade showed close correlation with the long-term OS [16, 61, 62], which have been reported in previous studies. Therefore, in order to enhance the predictive capability of the DLRS, we built the NHSTM-R model for outcome prediction by incorporating the DLRS and significant clinicopathological features. The NHSTM-R model showed significantly better discrimination than the DLRS, SSIGN and UISS scores. If the patients are stratified as high-score by the NHSTM-R model, intensive surveillance and systemic therapy are advocated. On the contrary, only regular surveillance is recommended for the low-score patients.

The present study has some limitations. Firstly, the samples size of our study was not large, this study should be validated in a multi-center prospective cohort. Secondly, although we explored the underlying association between radiomics features and prognosis, further validation was necessary.

## Conclusion

In conclusion, we developed a combined model for the stratification and prognostic prediction of ccRCC patients by incorporating the DL radiomics features and clinicopathological features. Radiomics could be used for individualized prognosis estimations by reflecting the differences in the tumor heterogeneity and microenvironment.

## Electronic supplementary material

Below is the link to the electronic supplementary material.

Supplementary Material 1

## Data Availability

No datasets were generated or analysed during the current study.

## Abbreviations

CT: Computed tomography
ccRCC: Clear cell renal cell carcinoma
DL: Deep learning
TCIA: The Cancer Imaging Archive
TCGA: The Cancer Genome Atlas
OS: Overall survival
DFS: Disease-free survival
ROI: Regions of interest
DLRS: Deep learning radiomics score
LASSO: Least absolute shrinkage and selection operator
ICCs: Intra- and inter-class correlation coefficients
AUC: Area under the curve
ROC: Receiver operating characteristic
IQR: Interquartile range
SHAP: SHapley Additive explanation
SSIGN: Stage, Size, Grade, and Necrosis
UISS: University of California, Los Angeles, Integrated Staging System
ECOG-PS: Eastern Cooperative Oncology Group
CCI: Charlson Comorbidity Index
WHO/ISUP: World Health Organization/International Society of Urologic Pathologists
XGBC: eXtreme Gradient Boosting classification
DCA: Decision curve analysis
DEG: Differentially expressed gene
GO: Gene Ontology
KEGG: Kyoto encyclopedia of Genes and Genomes
TMB: Tumor mutation burden
ITH: Intratumor heterogeneity
Treg: T regulatory cell
