# Supplementary material for: Radiomics predicts the prognosis of patients with clear cell renal cell carcinoma by reflecting the tumor heterogeneity and microenvironment
Source: Cancer Imaging. 2024 Sep 16;24:124. doi: 10.1186/s40644-024-00768-7 (PMC11403861; doi:10.1186/s40644-024-00768-7)
Supplement: Supplementary file 1 — Supplementary Material 1 [file 40644_2024_768_MOESM1_ESM.docx]

**Supplementary material**

**Supplementary Table S1.** **CT scan protocols**

|  | Hospital 1 | Hospital 2 | Hospital 3 |
| --- | --- | --- | --- |
| CT scanner | 64-slice | 64-slice | 64-slice |
| Manufacturer | Philips | Siemens | Siemens |
| Tube voltage (kV) | 120 | 120 | 120 |
| Tube current (mA) | 250 | 200 | 200 |
| Matrix | 512×512 | 512×512 | 512×512 |
| Slice thickness | 5 mm | 5 mm | 5 mm |
| Format | dicom | dicom | dicom |

**Supplementary S2. The SSIGN and UISS scores calculation**

The SSIGN score was calculated as 2 (pT1b) + 3 (pT2 tumors) + 4 (pT3a) + 4 (pT3b, pT3c, and pT4) + 2 (pN1 and pN2) + 1 (tumor size ≥10 cm) + 1 (grade 3) + 3 (grade 4)

+ 1 (necrosis), and 0 otherwise. Patients with 0 to 2 scores are considered low risk, those with 3 to 5 scores are considered intermediate risk, and patients with 6 or more scores are considered high risk.

The UISS was assessed according to the following criteria: patients with T1 stage, Fuhrman grade 1-2 and ECOG-PS 0 are considered low risk, those with T3 stage, grade 2-4 and ECOG- PS ≥1 or T4 stage are considered high risk, and the left patients are considered intermediate risk.

**Supplementary S.3. Model parameters**

XGBC, number of trees, 46; max_depth, 6; learning rate, 0.001; subsample, 0.2; colsample_bytree = 0.2.


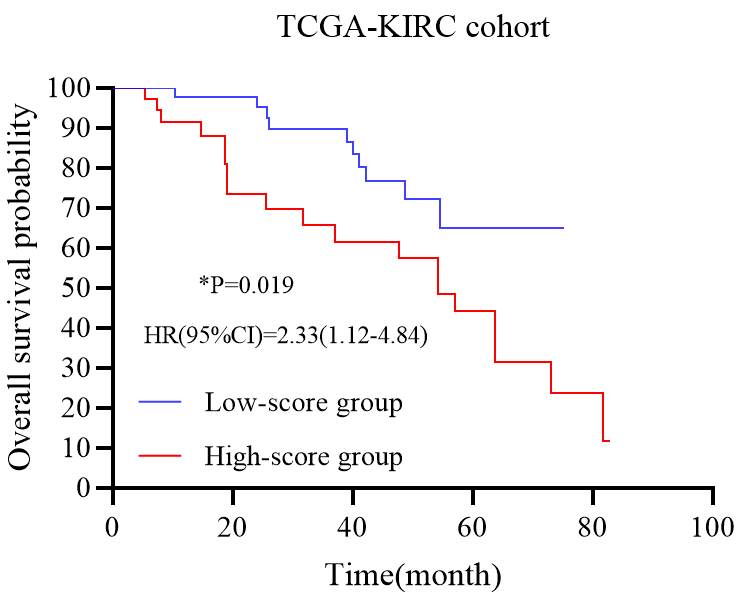


**Supplementary Figure S1.** Kaplan-Meier survival curves for overall survival in TCGA-KIRC cohort. Patients were stratified into high- (red line) and low -score (blue line) groups by the deep learning radiomics score(DLRS).


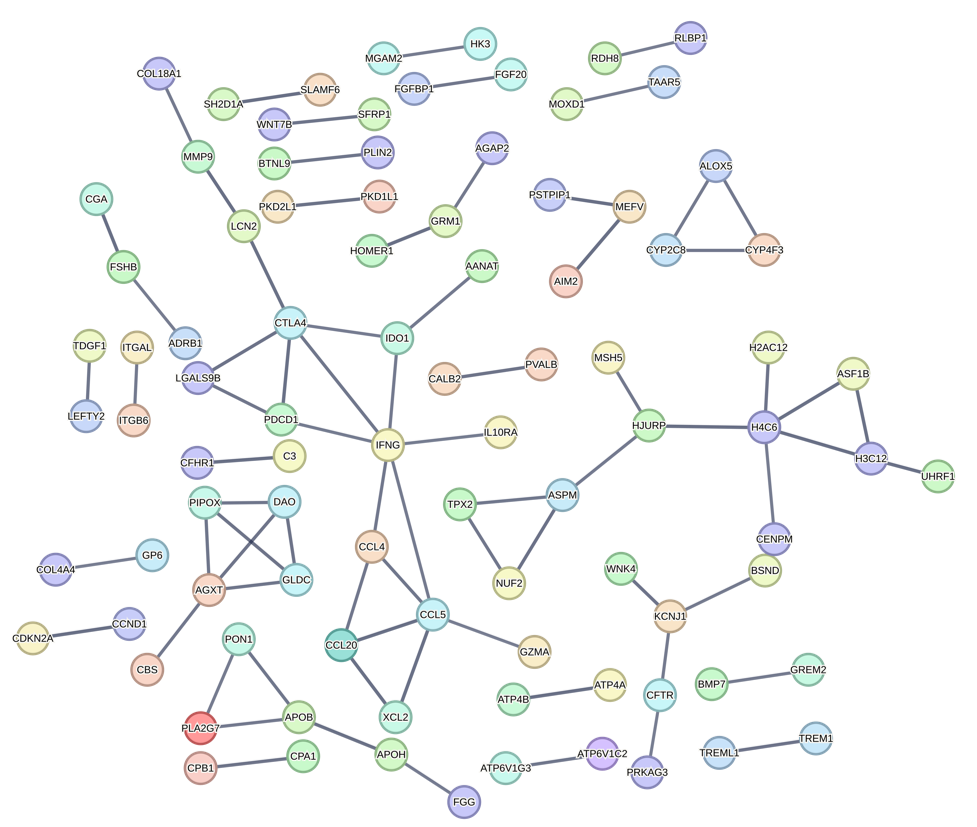


**Supplementary Figure S2.** The protein-protein interaction network of differential expression genes. A confidence score of ≥0.9 was set as the cutoff criterion.


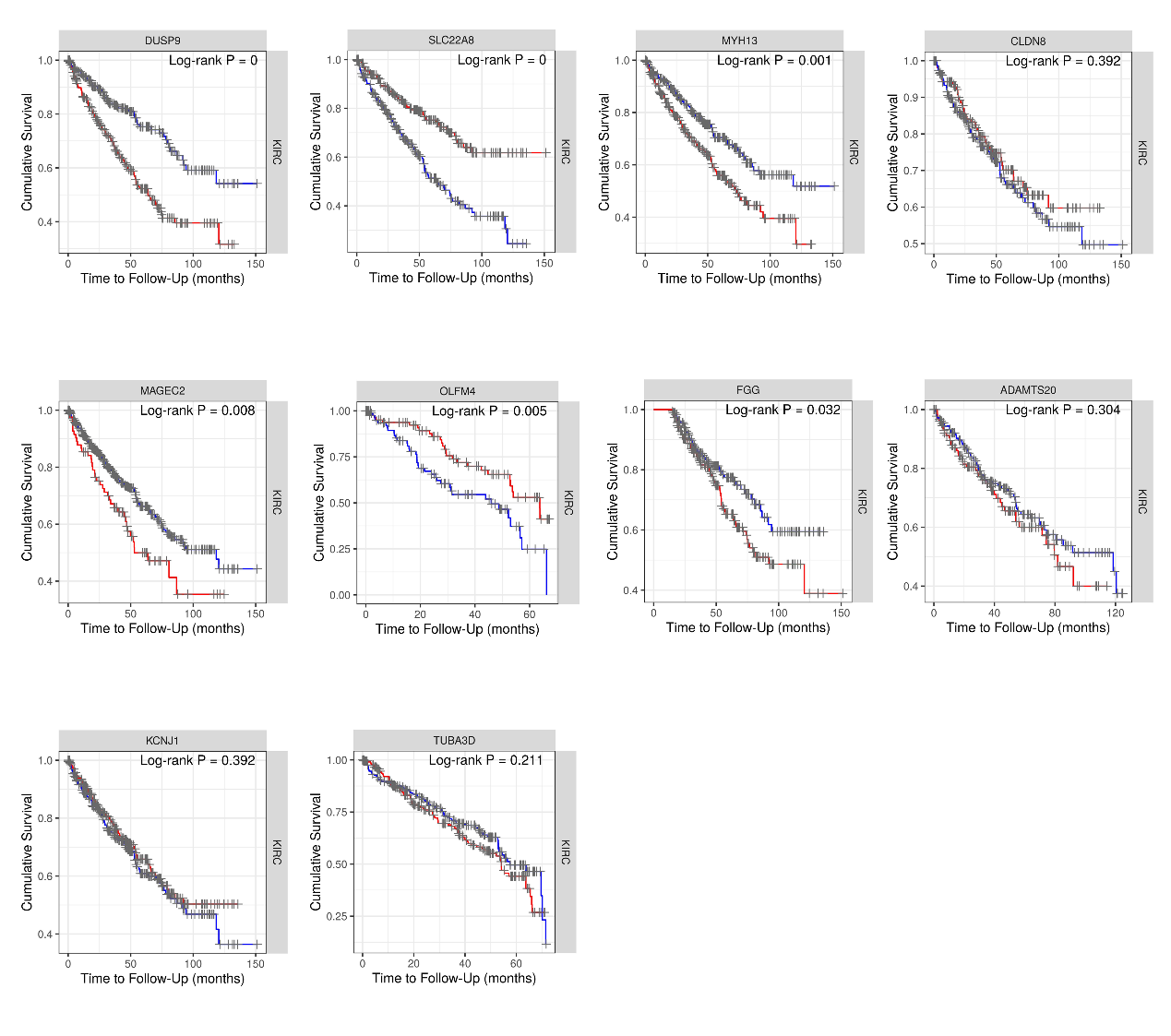


**Supplementary Figure S3.** The prognostic ability of the 10 most significant differential expression genes for overall survival. Kaplan–Meier analysis of overall survival in the TCGA‐KIRC cohort stratified by the deep learning radiomics score.


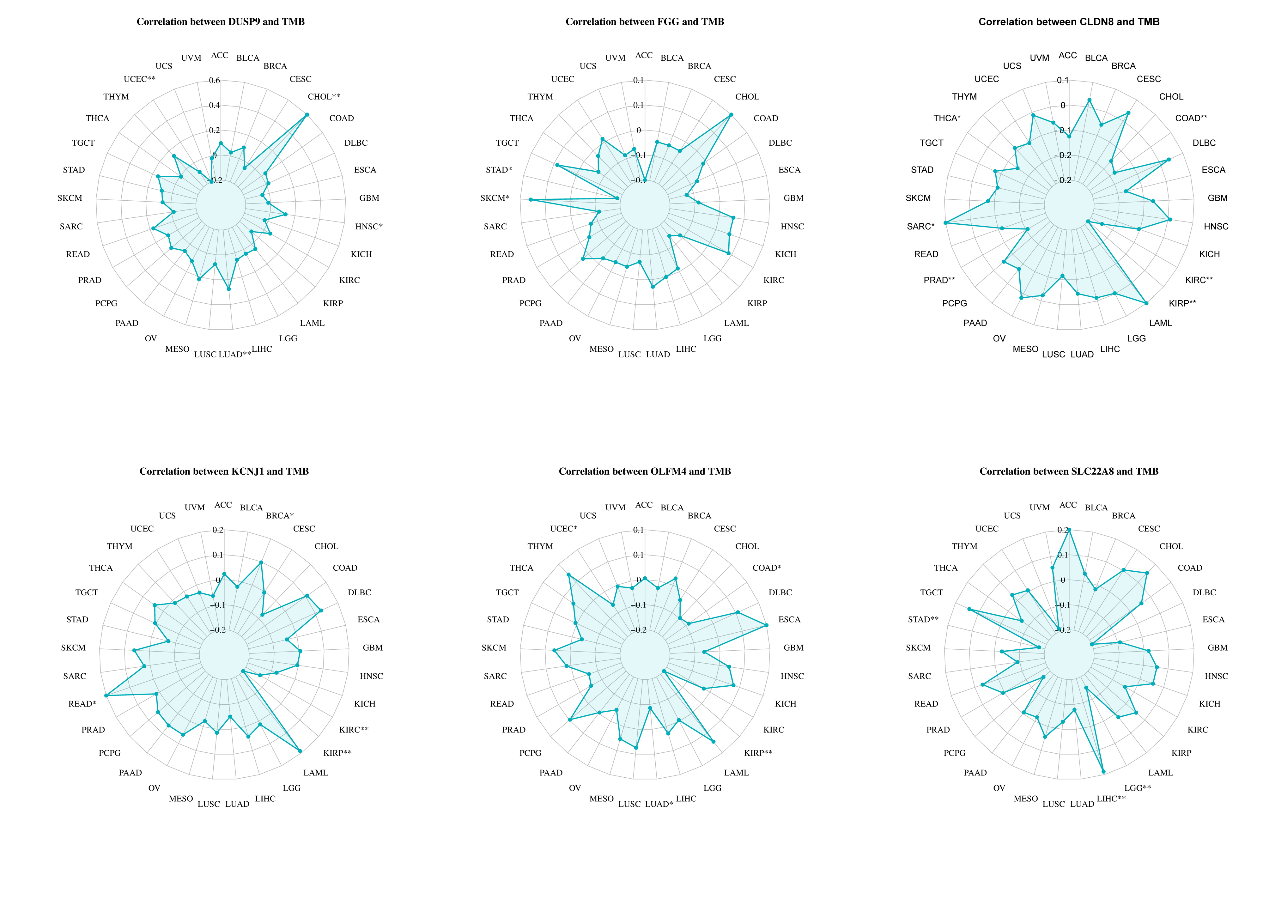


**Supplementary Figure S4.** The correlation between single gene expression and tumor mutational burden (TMB) was analyzed and the results were visualized with radar chart. CLDN8 and KCNJ1 gene expression showed significantly negative correlation with TMB of kidney renal clear cell carcinoma (KIRC) (p values<0.05).


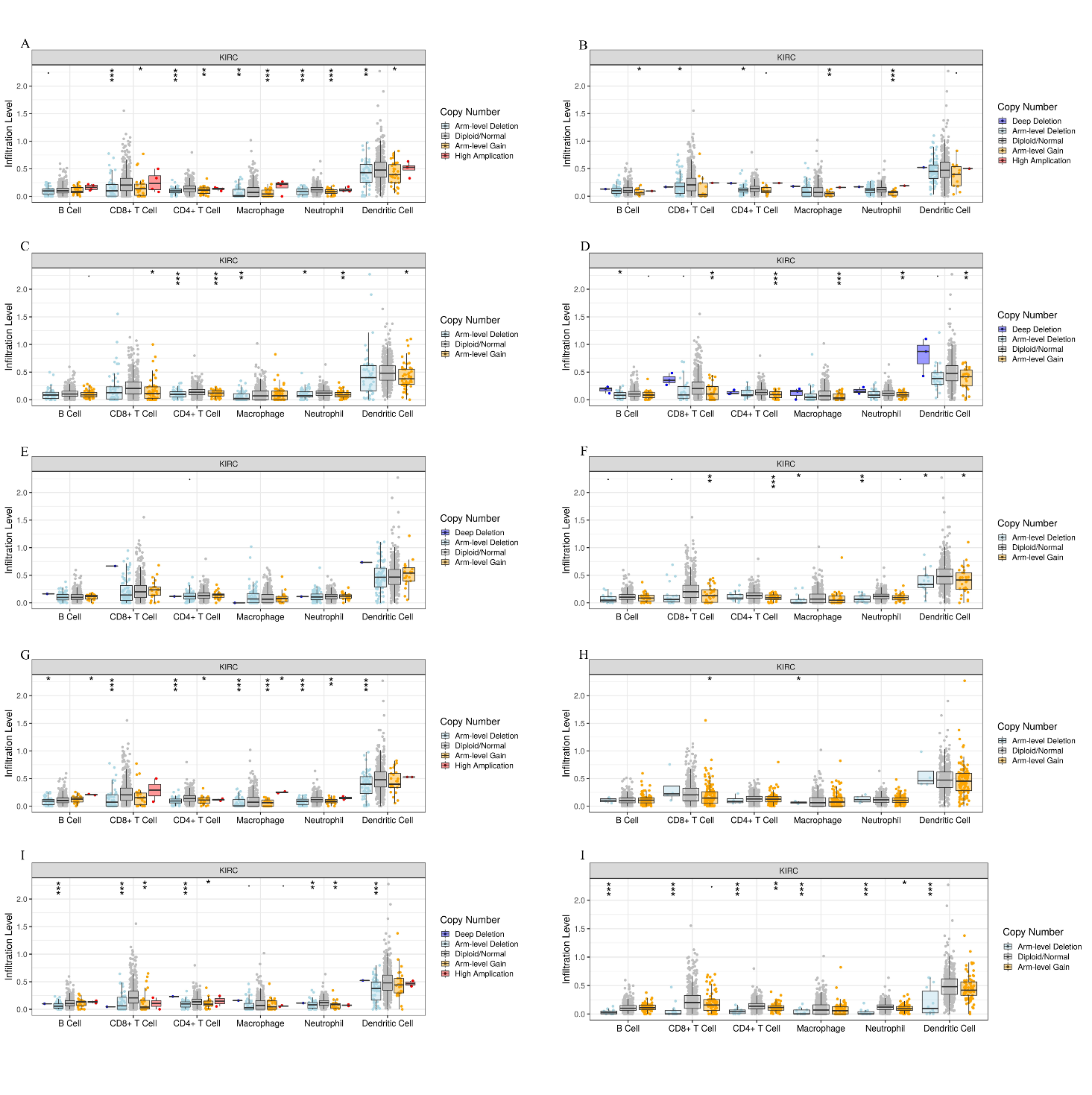


**Supplementary Figure S5.** The comparison of tumor infiltration levels among tumors with different somatic copy number alterations (SCNA) for the 10 most significant differential expression genes. Box plots are presented to show the distributions of each immune subset at each copy number status in kidney renal clear cell carcinoma (KIRC). The infiltration level for each SCNA category is compared using a two-sided Wilcoxon rank-sum test. (A) DUSP9 (B) FGG (C) CLDN8 (D) KCNJ1 (E) OLFM4 (F) SLC22A8 (G) MAGEC2 (H) ADAMTS20 (I) MYH13 (J) TUBA3D.
